# From Ideals to Tools: Applying Human Rights to Maternal Health

**DOI:** 10.1371/journal.pmed.1001546

**Published:** 2013-11-05

**Authors:** Alicia Ely Yamin

**Affiliations:** Program on the Health Rights of Women and Children, Franςois-Xavier Bagnoud Center for Health and Human Rights, Harvard University, Boston, Massachusetts, United States of America

## Abstract

Alicia Yamin argues that applying human rights frameworks and approaches to maternal health offers strategies and tools to address the root causes of maternal morbidity and mortality within and beyond health systems, in addition to addressing other violations of women's sexual and reproductive health and rights.

*Please see later in the article for the Editors' Summary*

**Summary Points**

- Applying human rights frameworks and human rights-based approaches (HRBAs) to maternal health offers strategies and tools to address root causes of maternal morbidity and mortality (MMM) within and beyond health systems, as well as other violations of women's sexual and reproductive health and rights (SRHR) across their lives, including poverty, gender inequality, and structural violence against women, rather than simply promoting short-term technical fixes.
- Lessons post-International Conference on Population and Development (ICPD) and Beijing, as well as the efforts of the last decade, show the importance of operationalizing HRBAs in order to convert aspirational ideals into actionable tools. Operationalization can and must take many forms, including social accountability efforts, as well as judicial enforcement; however, advancing SRHR requires changing decisions at multiple levels, not merely providing redress in the event of violations.
- The ultimate goal of adopting an HRBA is to offer strategies for addressing the underlying power relations that systematically put women—some more than others—at risk of SRHR violations, and MMM in particular, and enabling women to live lives of dignity.

## Introduction

In recent years, growing attention has been paid to human rights-based approaches (HRBAs) to health and development issues, such as HIV/AIDS, water and sanitation, and child health, not only from non-governmental organizations, but also from UN bodies [1]. Although diverse in nature, HRBAs are concerned not just with improving specific outcomes, but with transforming the underlying conditions that drive distributions of disease, and deprivations of rights. Interest in applying human rights frameworks and HRBAs to maternal health offers strategies and tools to address root causes of maternal morbidity and mortality (MMM) within and beyond health systems, as well as other violations of women's sexual and reproductive health and rights (SRHR) across their lives, including poverty, gender inequality, and structural violence against women, rather than simply promoting technical fixes [2],[3]. This article outlines achievements with respect to applying human rights frameworks to MMM and SRHR, and argues that recent efforts at operationalization allow HRBAs to be relevant in shaping decisions policymakers face.

## Establishing MMM as a Rights Issue: Changing Thinking

Under international law, it was always clear that an array of civil and political, as well as economic and social, rights were relevant to women surviving childbirth [4]. However, it was not until the mid-1990s that a growing consensus emerged in public health that the majority of obstetric complications are neither predictable nor preventable and that, therefore, rather than identify high-risk pregnancies, the key to addressing MMM was to provide access to skilled birth attendance and emergency obstetric care for all, as well as family planning [5]. This public health consensus—although far from universally implemented in practice—was an essential step in the establishment of applying human rights standards to maternal health. Just as HIV/AIDS activism spawned litigation and claims for effective anti-retrovirals beginning in the mid-1990s, it was painfully evident that women were dying as a consequence of specific governmental failures. Thus, clear obligations, duty-bearers, and remedies could be identified as a matter of international human rights law [6]. Nevertheless, from the beginning, human rights advocacy relating to maternal health looked beyond medical care; the structural discrimination and deprivations of rights affecting women throughout their lives remained central to applications of HRBAs.

Although there had been significant advocacy in the women's health movement previously, it was also in the mid-1990s that women's rights activists began to coalesce around an agenda for SRH, including maternal health, which included both autonomy and access to services as explicit human rights claims under international law [7],[8].

Building on the 1993 Vienna Conference on Human Rights, at which the international women's movement had made substantial progress with regard to the enshrinement of women's rights under international law, in the International Conference on Population and Development (ICPD) Programme of Action, women were recognized as subjects of decisions about their bodies and lives, and not objects of health and development programs. One year later, in Beijing, women's health was recognized as “determined by the social, political and economic context of their lives, as well as by biology” [8]. Thus, the promotion of women's health required restructuring societal power relations, together with laws and policies, in addition to medical responses.

Yet it proved more complicated to translate the shift in thinking from ICPD and Beijing into shifts in decision making on the ground than many in the women's health and rights movement had anticipated. Mainstream development communities were preoccupied with waning foreign aid levels and hortatory calls for structural transformation and gender equality were difficult to embed in and across national-level programs on the ground [9]. Many programs were simply re-packaged without changing underlying approaches [6],[10].

**Figure 1:**
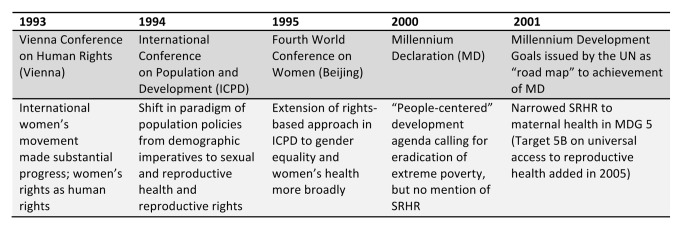
Timeline of relevant international conferences.

In the lead-up to the Millennium Declaration in 2000, there was also a significant political backlash from several forces at the global level, ranging from re-alignments in the G-77, to pressures from the Holy See, conservative Islamic states, and evangelical Christians in the United States [10],[11]. Moreover, the complexity of messaging around the need to address intersecting inequalities and social structures that affected women's health and rights did not fit into the Millennium Development Goal (MDG) agenda, with its narrow focus on outcomes. The broad agendas of ICPD and Beijing were reduced to the relatively depoliticized realm of maternal health in MDG5. With few exceptions, the international women's movement perceived the MDGs to be a betrayal of ICPD and Beijing commitments [12]. It was only in 2005 that MDG 5B was added, calling for “universal access to reproductive health,” and MDG 5B has been among the most lagging of targets (Figure 1).

## Achievements of HRBAs in the Context of Maternal Health

Applying human rights frameworks and tools to maternal health in many ways became the Trojan horse into which to fit SRHR during the MDGs. While some organisations that advanced HRBAs in the context of MDG 5 and maternal health had been actively working on SRHR for years, new actors joined the fray, including mainstream human rights groups, such as Amnesty International. The UN Special Rapporteur on the right to health played a pivotal role in highlighting maternal mortality as a human rights issue in his reports [13],[14]. What united efforts from both advocacy and service delivery organizations was a concern for combating intersecting forms of discrimination faced by women, promoting accountability, and providing women with a meaningful voice with respect to their SRH.

Although forcing women's health rights into strategies to achieve MDG 5 was frequently deplored as “instrumentalising” and “depoliticising” the SRHR that had been hard won at ICPD and Beijing, a critical mass of interest in maternal health as a human rights issue produced cutting-edge advocacy, including work on budgetary, economic, and fiscal policies, as well as engaging broader, non-traditional human rights constituencies within the health domain [15]–[17].

By 2013, the efforts of this collective advocacy on maternal mortality as a human rights issue were evident. Multiple fact-finding reports on MMM in different countries had brought to bear pressure on governments, achieving some notable victories in terms of changes in policies [18]. Social accountability strategies—including accompaniment of women to health facilities, use of crowd sourcing, and citizen budget analysis—had been mounted, enabling citizens to monitor their own health facilities and channel demands for change through district-level policymaking authorities [15],[19],[20].

Landmark litigation had been brought at both domestic and international levels, establishing important precedents regarding the obligations of governments to provide reproductive health care, including mandating expenditures and the exercise of due diligence with respect to private actors, as well as access to safe abortions [21]–[24]. At the time of writing, there are dozens of pending cases in India alone regarding governmental obligations to make maternal health services accessible to indigent women. Even in cases where litigation was not immediately successful in court, such as Uganda, it has mobilized public opinion around maternal health [25]. Although courts are generally weak actors, allies in country as well as in international organizations and networks have sustained pressure for implementation of major judgements.

At the UN level, the Human Rights Council (the Council) issued two historic resolutions regarding maternal mortality, which first established the normative connections between maternal mortality, SRH, and human rights, and in 2012 adopted a ground-breaking resolution regarding an HRBA in the context of MMM, which should serve as guidance in state reporting under the Universal Periodic Review (UPR) procedure before the Council [26],[27]. Under UPR, all countries, and not merely those that affirmatively ratify a specific treaty, are required to report on human rights obligations at the Council.

Moreover, failures of accountability and need for human rights protections were noted in the 2010 UN Secretary-General's Global Strategy on Women and Children, which specifically called upon the WHO to chair an “accountability process” on women's and children's health to implement the Global Strategy [28]. That process became the WHO Information and Accountability Commission on Women's and Children's Health [29], which emphasized human rights, especially in the context of promoting accountability, and led to the creation of an independent Expert Review Group (iERG) to report annually between 2012 and 2015 to the UN Secretary-General. Although it is too early to tell what impacts on accountability the iERG will have, it has made human rights a central part of its work.

## Operationalizing HRBAs: Changing Decisions

Lessons post-ICPD and Beijing, as well as the efforts of the last decade, show the importance of operationalizing HRBAs in order to convert aspirational ideals into actionable tools. Operationalization can and must take many forms, including the social accountability efforts mentioned above, as well as, critically, judicial enforcement [23]. However, advancing SRHR requires changing decisions at multiple levels, not merely providing redress in the event of violations. Therefore, policy guidance to governments is essential to answer the “so what?” question—i.e., how is an HRBA different from a conventional approach to decision making?

The adoption of Technical Guidance by the HRC in 2012 is a promising step in this regard [26]. This Technical Guidance, on which this author served as lead consultant, provides operational guidelines for policymakers on how to implement policies and programs to reduce MMM in accordance with human rights standards. However, the Technical Guidance should also be useful for courts, National Human Rights Institutions, and civil society advocates [26],[30].

The Technical Guidance reiterates that an HRBA requires addressing MMM in the broader framework of SRHR. It notes measures needed to address the social determinants of women's health, and emphasizes that HRBAs require multi-sectoral planning and budgeting processes [26], which are rarely the norm in conventional health planning in many countries.

Focused in particular on the health system as a core social institution, the Technical Guidance illustrates how adopting an HRBA should influence decisions at every stage of decision making from the initial situational analysis, and design of a national strategy and plan of action on SRH, to specifics on budget formulation and implementation, to programme implementation, to monitoring and evaluation, with the specific aim of creating a circle of accountability. In so doing, the Technical Guidance moves away from declaiming abstract principles to illustrating ways in which an HRBA calls for a different ethical calculus in decision making. For example, in an HRBA, budgetary allocations should account for patterns of historical discrimination and intersecting inequalities, rather than merely be targeted at producing greatest aggregate advances. Similarly, in monitoring and evaluation, quantitative data need not only to be disaggregated to reveal potential disparities and discrimination, but additionally indicators need to be selected that can measure compliance with international human rights obligations [26]. The Guidance also underscores the need for effective remedies, and spells out obligations of development partners with respect to financial assistance and policy coherence [26].

Implementation of this Technical Guidance at the national level, as well as its use in countries' mandatory UPR reporting can prove an important precedent not only for SRH, but also for the operationalization of HRBAs to other development issues.

## Conclusions

As the world reflects on the MDGs, and is poised to adopt a new development agenda, HRBAs offer strategies for addressing the underlying power relations that systematically put women—some more than others—at risk of SRHR violations, and MMM in particular. Not only is strategic litigation being combined with grassroots mobilization to demand reproductive health care in multiple countries [21],[23]–[25], but academia and activism around maternal health as a rights issue has pushed the boundaries of human rights strategies to address social and gender justice. Recent initiatives to operationalize HRBAs in the context of SRH and maternal health, including the UN Technical Guidance, are overdue and essential for the broader public health community to see human rights as useful tools that are relevant to complex policy trade-offs. Fostering operationalization of HRBAs is also critical to show concretely how global Goals can be translated through rights-based practices at a national level, and, in turn, to ensure the central importance of SRHR to the post 2015 development framework. Much is at stake beyond the achievement of MDG 5. The ultimate goal of adopting an HRBA in the context of SRH is not merely the avoidance of MMM, but enabling women to live lives of dignity. This goalis both the challenge, and the promise.
